# Prediction of Neurological Outcomes in Elderly Patients With Head Trauma Using the Geriatric Trauma Outcome Score: A Retrospective Observational Study

**DOI:** 10.7759/cureus.66768

**Published:** 2024-08-13

**Authors:** Yuta Iizawa, Yosuke Hayashi, Daiki Saito, Kengo Kondo, Mana Yamashiro, Rie Kanematsu, Kimihito Hirose, Michio Nakamura, Tadashi Miyazaki

**Affiliations:** 1 Department of Emergency and Critical Care Medicine, Chiba University Graduate School of Medicine, Chiba, JPN; 2 Department of Emergency and Critical Care Medicine, Japan Red Cross Narita Hospital, Narita, JPN; 3 Department of Neurosurgery, Japan Red Cross Narita Hospital, Narita, JPN

**Keywords:** cerebral performance category, geriatric trauma outcome score, neurological outcome, outcome prediction, head trauma, elderly patients

## Abstract

Introduction

Head trauma in elderly people is a problem in today's aging society. Elderly people are susceptible to head trauma because of their declining physical function; this tends to be severe. Outcome prediction is important in decision-making regarding treatment strategies; however, there is no unified method for predicting neurological outcomes in elderly patients with head trauma.

Methods

Elderly patients with head trauma admitted to the Japan Red Cross Narita Hospital between January 2019 and August 2023 were enrolled in this single-center, retrospective observational study. A favorable neurological outcome was defined as a cerebral performance category scale of 1 or 2. Multivariate logistic regression analysis and receiver operating characteristic curve analysis were performed to investigate the association between geriatric trauma outcome scores and outcomes and to evaluate the predictive value of geriatric trauma outcome scores. The primary outcome was a favorable neurological outcome at discharge, and the secondary outcome was in-hospital mortality.

Results

A total of 313 elderly patients with head trauma were eligible for analysis. Multivariate logistic regression analysis revealed that the geriatric trauma outcome score was significantly associated with a favorable neurological outcome at discharge (odds ratio 0.94, *P *<0.0001). In the receiver operating characteristic curve analysis, the geriatric trauma outcome score had a good predictive value for favorable neurological outcomes at discharge (area under the receiver operating characteristic curve 0.83).

Conclusions

The geriatric trauma outcome score had good predictive value for favorable neurological outcomes at discharge in elderly patients with head trauma and has the potential to aid in decision-making regarding treatment strategies for elderly patients with head trauma.

## Introduction

Population aging is prevalent in Japan and globally [1]. Trauma is an important issue in today’s aging society, as the decline in physical function seen in elderly people leads to trauma [2,3]. Once trauma has occurred, mortality in elderly patients is higher and the ability to perform activities of daily living (ADL) in survivors is lower [2] than in younger patients. The most common site of injury excluding the extremities is head trauma in elderly patients [3]. The incidence of head trauma, especially traumatic brain injury, which is the most severe form of head trauma, is increasing among elderly patients globally [4,5], and the outcomes of head trauma are poorer than those in younger patients [6]. Thus, head trauma in older adults is a major problem in today's aging society.

Outcome prediction is important in decision-making for treatment strategies; hence, several predictors have been reported, such as acute subdural hematoma (A-SDH) [7-9], oral anticoagulants [10-13], Glasgow coma scale (GCS) [9,14,15], and severity scores [16,17] for patients with head trauma. However, there is no unified method for predicting neurological outcomes in elderly patients with head trauma.

The injury severity score (ISS) is commonly used for outcome prediction for patients with trauma [18,19]. However, elderly patients with trauma differ from younger patients in that mortality in elderly patients is higher than that in younger patients, even with similar ISS [20], and the geriatric trauma outcome score (GTOS) was developed to predict mortality in elderly patients with trauma [21]. GTOS was first reported by Zhao et al. in 2015 and can be calculated at the bedside using only three factors: age, ISS, and transfusion of packed red blood cells (PRBCs). There have been few reports examining morbidity, but only one report investigated the association between GTOS and morbidity at discharge in elderly patients with trauma [22]. Whether GTOS is associated with neurological outcomes in elderly patients with head trauma is unclear.

We hypothesized that GTOS could predict neurological outcomes at discharge in elderly patients with head trauma. To demonstrate this, we analyzed the association between GTOS and neurological outcomes at discharge in elderly patients with head trauma who were admitted to our hospital.

## Materials and methods

Study setting and patients

This single-center, retrospective observational study was conducted at a tertiary emergency and critical care center of Japan Red Cross Narita Hospital, located in an urban area of Japan. The study was conducted from January 2019 to August 2023. Patients who visited the emergency department (ED) within the study period were screened for eligibility. We included elderly (≥65 years old) patients who were admitted to our hospital with the diagnosis of head trauma. We excluded patients who died in the ER or were diagnosed with a chronic subdural hematoma (C-SDH) or nontraumatic hemorrhage. If patients had been admitted multiple times, only first-admission data were analyzed.

The protocol for this research project was approved by a suitably constituted Ethics Committee of the institution and it conforms to the provisions of the Declaration of Helsinki (Committee of the Japan Red Cross Narita Hospital Certified Clinical Research Review Board, Approval No. 884-01). The requirement for written informed consent was waived by the Review Board because of the retrospective study design, in conformity with the Ethical Guidelines for Medical and Health Research Involving Human Subjects in Japan. Opt-outs for the study were posted on hospital websites.

Data collection and definition

Patient data were retrospectively reviewed and extracted from medical records. The collected data for analysis included patient characteristics (age, sex, mechanism of injury (traffic accident, fall, and others), and oral anticoagulants), type of cerebral lesions (traumatic subarachnoid hemorrhage (SAH), acute SDH (A-SDH), acute epidural hematoma (A-EDH), mixed lesions, and other hemorrhage, contusions, concussion, and others), severity scores (abbreviated injury score (AIS), ISS, and GTOS), and outcomes (mortality, length of hospital stay, and cerebral performance category (CPC) scale [23] and Glasgow outcome scale (GOS) [24] at discharge). The injury type was diagnosed by neurosurgeons during hospitalization and extracted based on the International Classification of Diseases, 10th Revision. A favorable neurological outcome at discharge was defined as a CPC score of 1 or 2. For the calculation of ISS, the six body regions (head and neck, face, chest, abdomen, extremities, and external) are assigned an AIS of 0 to 5 depending on the severity of the injury. AIS 0 means uninjured and 5 means maximally injured while still being compatible with life. The ISS of a patient is calculated from the sum of the squares of the three worst AISs [18]. The GTOS was calculated using the following formula [21]:

GTOS = Age + (2.5*ISS) + 22 (if given PRBCs within 24 h of admission)

Table 1 lists the CPC scores.

**Table 1:** Cerebral performance category scores

Score		
1	Good Cerebral Performance (Normal Life)	Conscious, alert, able to work and lead a normal life. May have minor psychological or neurologic deficits (mild dysphasia, non-incapacitating hemiparesis, or minor cranial nerve abnormalities).
2	Moderate Cerebral Disability (Disabled but Independent)	Conscious. Sufficient cerebral function for part-time work in a sheltered environment or independent activities of daily life (dress, travel by public transportation, food preparation). May have hemiplegia, seizures, ataxia, dysarthria, dysphasia, or permanent memory or mental changes.
3	Severe Cerebral Disability (Conscious but Disabled and Dependent)	Conscious; dependent on others for daily support (in an institution or at home with exceptional family effort). Has at least limited cognition. This category includes a wide range of cerebral abnormalities, from patients who are ambulatory but have severe memory disturbances or dementia precluding independent existence to those who are paralyzed and can communicate only with their eyes, as in the locked-in syndrome.
4	Coma/Vegetative State (Unconscious)	Unconscious, unaware of surroundings, no cognition. No verbal or psychological interaction with the environment.
5	Brain Death (Certified brain dead or dead by traditional criteria)	Certified brain dead or dead by traditional criteria.

Statistical analysis

The primary outcome was a favorable neurological outcome (CPC ≤2) at discharge and the secondary outcome was in-hospital mortality. To clarify the association between the GTOS and outcomes, we performed univariate and multivariate logistic regression analyses, calculated the odds ratio (OR) and 95% confidence interval (CI), and compared them with the ISS and AIS scores of the head and neck. Multivariate analysis was adjusted by sex, mechanism of injury (traffic accident, fall, and others), type of cerebral lesions (traumatic SAH, A-SDH, A-EDH, mixed lesions, other hemorrhage, contusions, concussion, and others), and oral anticoagulants.

Model performance was measured using receiver operating characteristic curve (ROC) analysis, and the area under the ROC (AUROC) was evaluated. The cut-off value of the GTOS was calculated using the Youden index.

Moreover, we performed the above analysis using GOS, which is used as another outcome scale in previous studies [25], as good recovery (GOS=5) is a favorable neurological outcome. Furthermore, we performed a subgroup analysis according to the severity of head trauma. Patients were divided based on their AIS into a severe head trauma group (AIS=1,2) and a non-severe head trauma group (AIS ≥3).

Data are expressed as medians and interquartile ranges for continuous variables and absolute numbers (%) for categorical variables. The AUROC was expressed with CI. Statistical significance was set at P <0.05. Analyses were performed using the IBM SPSS Statistics for Macintosh, Version 26.0.0. (Armonk, NY: IBM Corp).

## Results

Baseline characteristics and clinical outcomes

**Figure 1:**
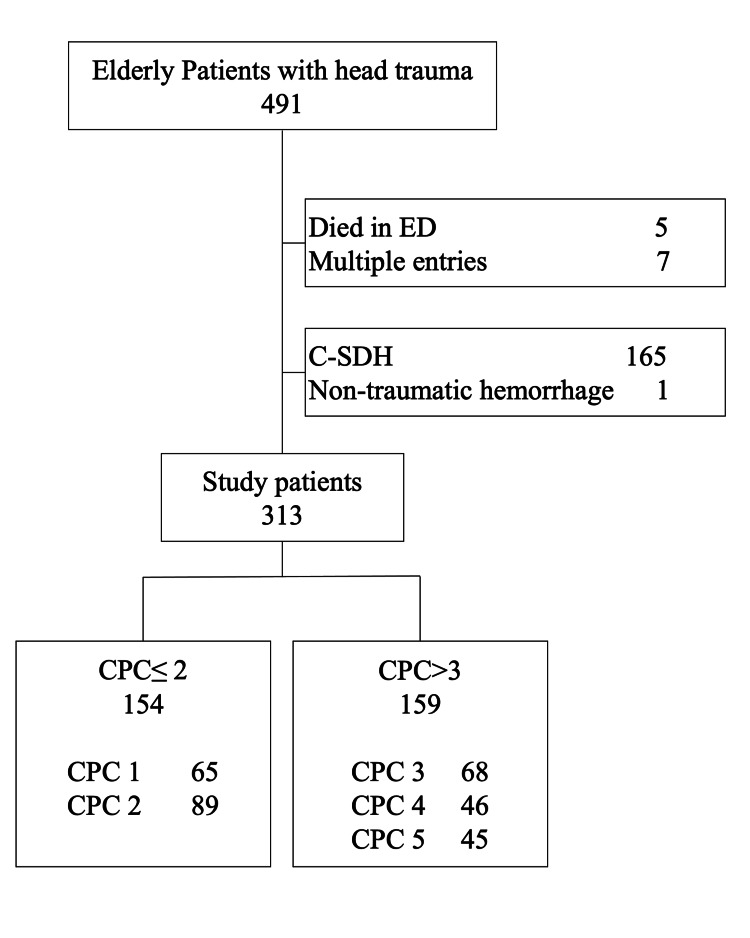
Patient flowchart ED, emergency department; C-SDH, chronic subdural hematoma; CPC, cerebral performance category.

A total of 491 elderly patients with head trauma were screened. Of these, 313 patients were analyzed after excluding 5 patients who died in the ED, 7 who had been admitted multiple times, 165 with a diagnosis of C-SDH, and 1 with a non-traumatic disease (Figure 1).

In total, 154 patients had a favorable neurological outcome at discharge (65 patients were CPC 1 and 89 patients were CPC 2) and were classified into the CPC ≤2 group. In contrast, 159 patients had an unfavorable outcome at discharge (68 patients were CPC 3, 46 patients were CPC 4, and 45 patients were CPC 5) and into the CPC >2 group. The CPC ≤2 group had a significantly lower age, a higher percentage of male patients, and falls as a mechanism of injury than the CPC >2 group. As the type of injury, the CPC >2 group had significantly higher traumatic SAH and concussion and lower A-SDH and mixed hemorrhage. AIS points of head and neck, ISS, and GTOS in the CPC ≤2 group were significantly lower in the CPC ≤2 group, and the length of hospital stay was significantly shorter in the CPC ≤2 group (Table 2). Patients who died were classified into the CPC >2 group according to the definition of CPC. In-hospital mortality was 28.3% in the CPC >2 group.

**Table 2:** Baseline characteristics and outcomes of the CPC ≤2 group and the CPC >2 group CPC, cerebral performance category; SAH, subarachnoid hemorrhage; A-SDH, acute subdural hematoma; A-EDH, acute epidural hematoma; GTOS, geriatric trauma outcome score; PRBCs, packed red blood cells Data are presented as median and interquartile range for continuous variables. P-values were calculated using the Mann-Whitney U test.

	CPC ≤2 (n = 154)	CPC >2 (n = 159)	P-value
Age, years	78.0 (72.0–82.0)	82.0 (76.0–87.0)	<0.001
Age group			
Pre-old, n (%)	57 (37.0)	32 (20.1)	<0.01
Old, n (%)	69 (44.8)	65 (40.9)	0.48
Oldest-old, n (%)	28 (18.2)	62 (39.0)	<0.001
Male sex, n (%)	81 (52.6)	112 (70.4)	<0.01
Mechanism of injury			
Traffic accident, n (%)	22 (14.3)	15 (9.4)	0.19
Fall, n (%)	97 (61.0)	116 (75.3)	<0.01
Unknown, n (%)	16 (10.4)	47 (29.6)	<0.001
Oral antithrombotic, n (%)	50 (32.5)	48 (30.2)	0.66
Type of injury			
Traumatic SAH, n (%)	48 (31.2)	28 (17.6)	<0.01
A-SDH, n (%)	35 (22.7)	72 (45.3)	<0.001
A-EDH, n (%)	7 (4.5)	11 (6.9)	0.37
Mixed hemorrhage	8 (5.2)	20 (12.6)	<0.05
Other hemorrhage	5 (3.2)	2 (1.3)	0.24
Contusion	15 (9.7)	23 (14.5)	0.20
Concussion	29 (18.8)	2 (1.3)	<0.001
Other	7 (4.5)	1 (0.6)	<0.05
Abbreviated injury score			
Head and neck	2.0 (2.0–3.0)	4.0 (3.0–5.0)	<0.001
Face	0.0 (0.0–0.0)	0.0 (0.0–0.0)	0.28
Chest	0.0 (0.0–0.0)	0.0 (0.0–0.0)	0.85
Abdomen	0.0 (0.0–0.0)	0.0 (0.0–0.0)	0.98
Extremities	0.0 (0.0–0.0)	0.0 (0.0–0.0)	0.10
External	0.0 (0.0–1.0)	0.0 (0.0–0.0)	<0.05
Injury severity score	8.0 (4.0–9.0)	17.0 (9.0–25.0)	<0.001
GTOS	94.8 (85.6–106.3)	126.0 (107.5–145.0)	<0.001
Transfusion of PRBCs, n (%)	1 (0.6)	1 (0.6)	0.98
Outcome			
Mortality, %		28.3	
Length of hospital stay, days	5.2 (2.3–9.0)	17.8 (3.9–34.0)	<0.001

The association between a favorable neurological outcome and the GTOS 

The univariate logistic regression analysis revealed the association between a favorable neurological outcome at discharge and scoring systems (GTOS, OR 0.94, CI 0.93-0.96, P <0.001; ISS, OR 0.86, CI 0.83-0.89, P <0.001; AIS points of the head and neck, OR 0.37, CI 0.29-0.46) (Table 3). In the multivariate analysis adjusted by sex, mechanism of injury, type of cerebral lesions, and oral anticoagulants, the GTOS was significantly associated with a favorable neurological outcome at discharge (OR 0.94, CI 0.92-0.96, P <0.001).

**Table 3:** Univariate and multivariate analysis for the association between outcomes at discharge and the GTOS, ISS, and AIS in elderly patients Multivariate analysis was adjusted by sex, mechanism of injury (traffic accident, fall, and others), type of cerebral lesions (traumatic subarachnoid hemorrhage, acute subdural hematoma, acute epidural hematoma, mixed lesions, other hemorrhage, contusions, concussion, and others), and oral anticoagulants. GTOS, geriatric trauma outcome score; ISS, injury severity score; AIS, abbreviated injury scale; CPC, cerebral performance category

	Odds ratio	P-value
Univariate		
CPC ≤2 at discharge		
GTOS	0.94 (0.93–0.96)	<0.001
ISS	0.86 (0.83–0.89)	<0.001
AIS (head and neck) ≧4	0.37 (0.29–0.46)	<0.001
Mortality		
GTOS	1.07 (1.05–1.09)	<0.001
ISS	1.25 (1.17–1.33)	<0.001
Multivariate		
CPC ≤2 at discharge		
GTOS	0.94 (0.92–0.96)	<0.001
ISS	0.86 (0.81–0.90)	<0.001
AIS (head and neck) ≧4	0.32 (0.22–0.47)	<0.001
Mortality		
GTOS	1.08 (1.05–1.11)	<0.001
ISS	1.31 (1.19–1.43)	<0.001

**Figure 2:**
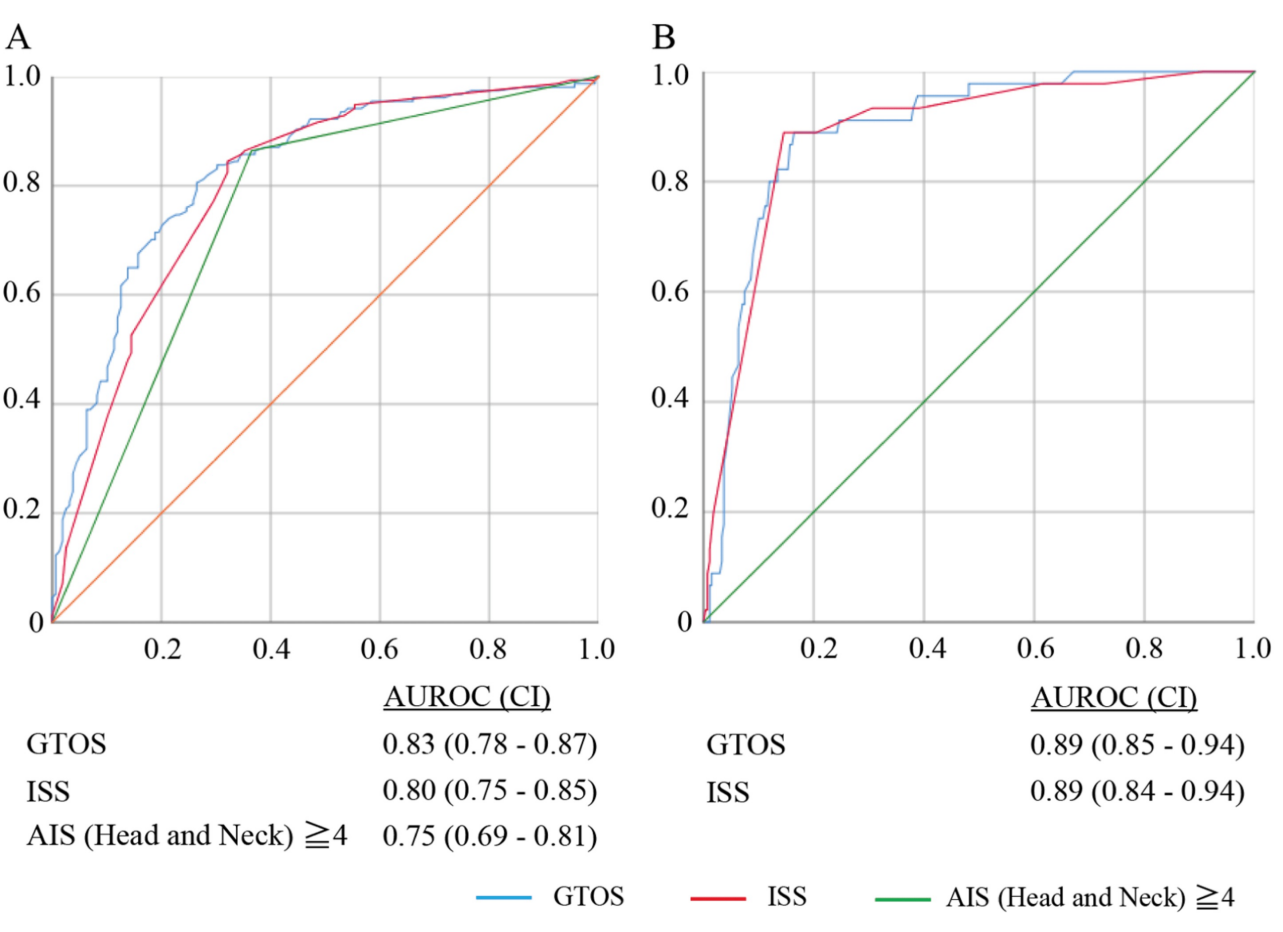
Receiver operation characteristic analysis of outcomes A. CPC ≤2 at discharge; B. Mortality AUROC, area under the receiver operation characteristic curve; CI, confidence interval; GTOS, geriatric trauma outcome score; ISS, injury severity score; AIS, abbreviated injury scale; CPC, cerebral performance category

In the ROC analysis, GTOS had the highest AUROC (GTOS, AUROC 0.83, CI 0.78-0.87; ISS, AUROC 0.80, CI 0.75-0.85; AIS points of head and neck, AUROC 0.75, CI 0.69-0.81) (Figure 2A). The cutoff value of the GTOS as a predictor of a favorable outcome at discharge was 107.5 according to the Youden index with a sensitivity of 0.76 and a specificity of 0.77.

**Figure 3:**
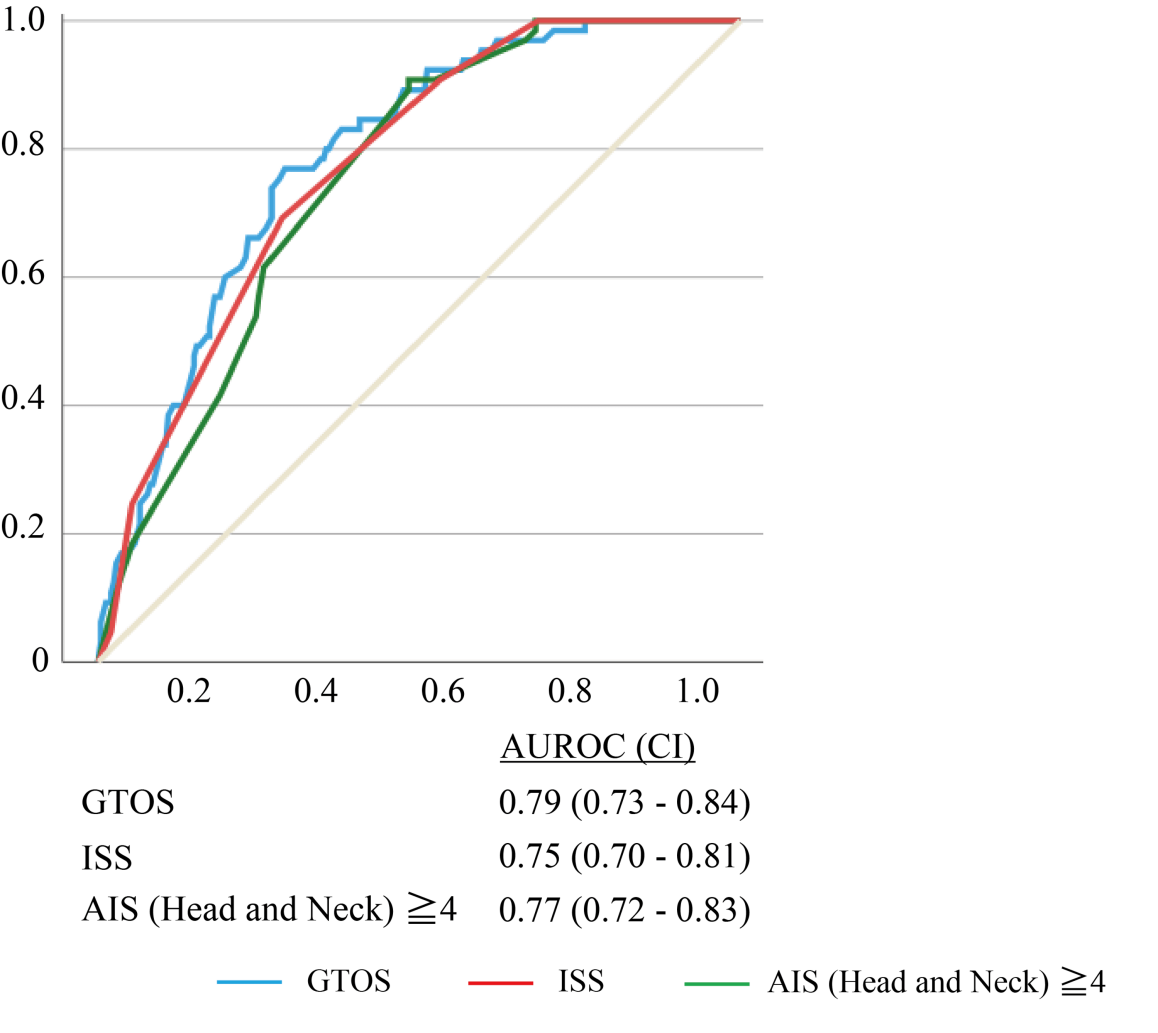
Receiver operation characteristic analysis of Glasgow Outcome Scale. AUROC, area under the receiver operation characteristic curve; CI, confidence interval; GTOS, geriatric trauma outcome score; ISS, injury severity score; AIS, abbreviated injury scale

Furthermore, even using the GOS as a neurological outcome at discharge, GTOS had the highest AUROC (GTOS, AUROC 0.79, CI 0.73-0.84; ISS, AUROC 0.75, CI 0.70-0.81; AIS points of head and neck, AUROC 0.77, CI 0.72-0.83) (Figure 3).

The association between mortality and the GTOS

The univariate logistic regression analysis revealed the association between mortality and severity scores (GTOS, OR 1.07, CI 1.05-1.09, P <0.001; ISS, OR 1.25, CI 1.17-1.33, P <0.0001) (Table 3). In the multivariate analysis, the GTOS was significantly associated with mortality (OR 1.08, CI 1.05-1.11, P <0.001). In the ROC analysis, the GTOS and ISS had almost equal AUROC (GTOS, AUROC 0.89, CI 0.85-0.94; ISS, AUROC 0.89, CI 0.84-0.94) (Figure 2B).

Subgroup analysis according to the severity of head trauma

Patients were divided into a severe head trauma group (n=197) and a non-severe head trauma group (n=116). In the subgroup analysis, the GTOS was significantly associated with a favorable neurological outcome at discharge defined as CPC ≤2 in both the severe (univariate, OR 0.95, CI 0.93-0.97, P <0.001; multivariate, OR 0.94, CI 0.92-0.96, P <0.001) and non-severe (univariate, OR 0.94, CI 0.90-0.99, P=0.015; multivariate, OR 0.95, CI 0.91-0.99, P=0.046) subgroups. In the ROC analysis, the GTOS had an acceptable performance in the severe group (AUROC 0.77, CI 0.70-0.85) and poor AUROC in the non-severe group (AUROC 0.67, CI 0.55-0.79).

## Discussion

The present study demonstrated that a higher GTOS was associated with worse neurological outcomes at discharge in elderly patients with head trauma. Furthermore, the GTOS had a high predictive value for neurological outcomes at discharge, which was higher than the ISS and AIS scores of the head and neck. The GTOS also had a high predictive value for in-hospital mortality, which was almost equal to the ISS. In addition, the subgroup analysis revealed that the GTOS was significantly associated with a favorable neurological outcome at discharge in both the severe and non-severe subgroups, and had acceptable AUROC in the severe subgroup. Therefore, the GTOS has the potential to be a tool for the outcome prediction precision of elderly patients with head trauma and can be a useful scoring system for decision-making. The valid cutoff values should be clarified through a multicenter prospective study in the future.

In today's aging population, it is important to recognize the characteristics of elderly people who are susceptible to trauma owing to a decline in various physical functions, such as the visual and musculoskeletal systems, and have slower reflexes and reaction times to avoid trauma [2,3]. In addition, having comorbidity and medication such as oral anticoagulants increases the risks of trauma [12,13,26,27]. In this study, the most frequent mechanism of injury was falling, probably due to a decline in physical function, and approximately one-third of patients were taking oral anticoagulants. These effects in elderly patients with trauma tend to be more severe, elderly patients with trauma have higher mortality rates than younger patients, even those with similar ISS [20]. Therefore, predicting the outcomes is important in elderly patients with trauma. This study focused on the GTOS, which was developed to predict mortality in elderly patients, and showed that it had a good predictive value for outcomes, including neurological outcomes, in elderly patients with head trauma. Additionally, the subgroup analysis showed a significant association between the GTOS and a favorable neurological outcome, and the GTOS had an acceptable AUROC in the severe head trauma group. In the non-severe subgroup, the GTOS was still significantly associated with a favorable neurological outcome; however, it had a poor predictive value. These findings suggest that the GTOS may be more beneficial in predicting the outcome of patients with severe head trauma.

Various risk factors of mortality and morbidity in patients with head trauma have been reported. In terms of cerebral lesions, A-SDH is known to be associated with a poor prognosis due to a high tendency to cause serious brain damage [7-9]. From the perspective of medication, oral anticoagulants are a risk factor for poor prognosis due to worsening bleeding [10-13]. In addition, some studies have shown that the GCS on admission is associated with mortality [14,15] and morbidity [9]. Cerebral injury is not only a brain disease, patients with brain injury may develop other organ failure even if they have no systemic diseases or infection [28]. In terms of a physiologic point of view, severity scores, such as the APACHE II score [17] or SOFA score [16], were associated with mortality. However, there are currently no established outcome prediction systems. In this study, GTOS showed a high predictive AUROC value of 0.83 for predicting a favorable neurological outcome and an AUROC of 0.89 for mortality. This study is valuable in revealing that the GTOS is useful for predicting outcomes in elderly patients with head trauma.

This study has several limitations. First, there may have been a selection bias because this was a single-center retrospective study. Numerous factors would affect the prognosis of patients with head trauma and that makes it incredibly difficult to predict outcomes of patients with head trauma. We performed the multivariate analysis adjusted sex, mechanism of injury, type of cerebral lesions, and oral anticoagulants; however, there were still a lot of factors that should be considered such as the volume of hematomas, prehospital process, and time course of physical rehabilitation. These were not able to be extracted accurately from the electronic medical record. The results of this study should be validated in a prospective multicenter study, including the determination of the appropriate cutoff value of GTOS for decision-making regarding treatment strategies. Second, our study patients had a few serious injuries to other parts of the body. It is well known that there is a relationship between head trauma and torso trauma, which can lead to serious conditions. Head trauma can cause severe hyperfibrinolysis [29,30], which leads to severe torso hemorrhage in patients with multiple trauma. Patients with severe multiple trauma, including head trauma, are an important research target and should be validated in these patient groups in the future. Third, few patients received PRBC transfusions. The GTOS is composed of three factors: age, ISS, and transfusion of PRBC; however, we could not investigate the effect of transfusion of PRBC on outcome prediction. This could be attributed to the small number of patients with severe multiple trauma, which should be validated in the future. Fourth, we did not consider cognitive impairment prior to being admitted to our hospital because of the lack of exact information from medical records. Age in the CPC >2 group was significantly higher, so they might have a higher previous impairment before injury. It may cause a bias.

## Conclusions

This is a single-center retrospective study investigating the prediction of neurologic outcomes at discharge in elderly patients with head trauma for which there have been no unified methods. This study revealed that the GTOS has good predictive value for favorable neurological outcomes at discharge in elderly patients with head trauma.

The incidence of head trauma is increasing in elderly patients globally and its severity is higher than in the younger. Improving the outcome of head trauma in elderly patients is a major challenge in today's aging society. The GTOS has the potential to be useful for predicting outcomes and aids in decision-making regarding treatment strategies for elderly patients with head trauma. As for future research, it is necessary to reveal appropriate cutoff values for the prediction in a multicenter prospective study and to establish treatment strategies based on GTOS for improving the neurological outcomes of head trauma in elderly patients.
